# Methadone Toxicity in a Pediatric Patient: A Case Report

**DOI:** 10.7759/cureus.14860

**Published:** 2021-05-05

**Authors:** Palveen Lumba, Marsha Medows, Hitesh Lumba, Leonita Bray

**Affiliations:** 1 Pediatrics, Woodhull Medical Center, New York, USA; 2 Pediatrics, New York University School of Medicine, New York, USA; 3 Pharmacology, Kharkiv National Medical University, Kharkiv, UKR

**Keywords:** methadone toxicity, mmt, urine drug testing, altered mental state, naloxone

## Abstract

Pediatric morbidity and mortality associated with methadone poisoning have been rising over the years. In this report, we discuss a case of a four-year-old boy who presented with respiratory distress and a depressed level of consciousness. A urine drug testing was performed given the miosis along with the depressed level of consciousness; the test was found to be positive for methadone, and no other cause of drowsiness was identified. This report highlights the importance of urine drug testing in pediatric patients presenting with a depressed level of consciousness.

## Introduction

There are several reported cases in the literature that highlight the dangers of methadone poisoning in pediatric patients. Methadone is a synthetic diphenyl propylamine that is well absorbed in the gastrointestinal tract. It is effective in treating nociceptive and neuropathic pain and is widely used in the management of opioid dependence [1]. The methadone maintenance treatment (MMT) program is a form of medication-assisted treatment that is being used to treat opioid use disorder under the Substance Abuse And Mental Health Services Administration (SAMHSA). As part of this program, carry-home dosages of methadone are dispensed to the patients in the form of oral tablets or oral solutions. This solution can be accidentally ingested by children, or some parents may add it to infant formula with the misguided intent of calming a fussy baby [2]. Even small doses of methadone can cause harmful effects in the opiate-naive pediatric population. In this report, we discuss a case of methadone poisoning in a four-year-old male patient.

## Case presentation

A previously healthy four-year-old boy was brought to our emergency by EMS at 9 pm with the chief complaint of difficulty in breathing. History provided by the child's mother (who had not been with him for three days) seemed inadequate and lacking in details. The initial interview revealed tactile fever for a day with a runny nose for which he had been treated with an unknown quantity of Tylenol PM (containing acetaminophen 500 mg and diphenhydramine 25 mg). The mother had noted that the patient had difficulty breathing upon arriving at her relative's home to pick him up. The patient's vitals were stable at presentation. He exhibited drowsiness but was responsive to loud voices and pain. He was observed to be in respiratory distress and stridorous. He was initially diagnosed with croup and managed accordingly but the cause of his drowsiness could not still be explained. Laboratory evaluation showed unremarkable complete blood count (CBC) and basic metabolic panel (BMP). Serum acetaminophen and serum salicylate levels were sent to rule out the respective toxicities but were found to be within normal limits.

A follow-up physical examination in the emergency department was significant for miosis and poorly responsive pupils. Urine toxicology was sent due to concerns regarding the patient's mental status and suspicion of drug ingestion. CT scan of the brain was performed due to a questionable fall documented in the patient's medical history; the CT findings were normal. The patient was admitted to the special care unit with pending urine toxicology screen. Urine toxicology was positive for methadone. The patient's mental state normalized before the urine results returned. Social work authorities were consulted regarding the disposition of the patient.

## Discussion

The patient's symptoms of altered mental status, lethargy, and miosis were consistent with opioid or methadone poisoning. According to a study published in an Iranian journal in 2015, clinical manifestations of methadone poisoning include drowsiness (91.4%), miotic pupils (75.9%), vomiting (69%), rapid shallow breathing (62.1%), and apnea (53.4%). This cross-sectional study included 58 children under the age of 12 years with a mean age of 5.2 ±1 years. All the cases in the study involved accidental poisoning caused by improper storage of the medication in water bottles and containers for other drugs. The classic triad of methadone poisoning, which includes central nervous system (CNS) depression, miosis, and altered breathing pattern, was reported in 53% of the cases [3].

A systemic literature review of 38 studies about methadone toxicity involving 62 children was published in 2012. Among these 62 reported cases, 29 died due to fatal ingestion. These fatal ingestions were associated with a higher serum methadone concentration ranging between 60-1,200 ng/ml (mean: 385 ng/ml); the children who survived had a lower serum methadone concentration in the range of 30-360 ng/ml (mean: 100 ng/ml) [4]. Another review article published data from a referral center in 2017; it included 453 cases and focused on the risk factors associated with death and intubation in methadone poisoning. Fever, elevated aspartate aminotransferase (AST) levels, prolonged QTc (>480 ms), and tachycardia were found to be independently associated with intubation and mortality [5]. The American Association of Poison Control tallied 2,186 cases of methadone exposure among children between the years 2000-2008 and found 20 reported deaths. The Drug Abuse Warning Network (DAWN) data from 2004 to 2011 have shown that an estimated 22,174 ED visits involved accidental ingestion of opioid pain relievers by children aged one to five years, out of which 941 involved methadone [6]. These publications and data point toward the dangerous and potentially lethal effects of methadone ingestion in the pediatric population.

Toxicology screen samples in children include urine, blood, saliva, and hair. Urine drug testing is the most popular among all the screens because of its easy availability. Naloxone is the preferred treatment for opioid overdose [7]. The use of one or two boluses of naloxone could help in the rapid establishment of diagnosis and be potentially life-saving [8]. Hence, infusion of naloxone should be considered in cases of life-threatening methadone toxicity. An observation period of 6-12 hours is recommended after stopping naloxone due to its short half-life.

## Conclusions

The number of methadone poisoning cases in the pediatric age group is on the rise due to the availability of methadone to parents or caregivers under the MMT program. Parents are often negligent about the harmful and potentially fatal effects of even small doses of methadone in children. Reported cases include accidental poisoning among young children and deliberate use of methadone in infants by the caregiver to “calm them”. Raising awareness among parents and caregivers under MMT regarding the potentially fatal effects of methadone and the importance of proper storage of the prescribed methadone solution could help to prevent accidental cases of poisoning among toddlers and children. Methadone poisoning should be considered in patients that present with CNS depression, miosis, and labored breathing, especially in cases where the history is unreliable. Naloxone use could be diagnostic and life-saving. Naloxone nasal spray could be provided as “at-home rescue kits” to patients in the MMT program, especially those residing in houses with children.
